# High Oncolytic Activity of a Double-Deleted Vaccinia Virus Copenhagen Strain against Malignant Pleural Mesothelioma

**DOI:** 10.1016/j.omto.2020.08.011

**Published:** 2020-08-25

**Authors:** Tiphaine Delaunay, Joelle Nader, Marion Grard, Isabelle Farine, Vera Hedwig, Johann Foloppe, Thibaut Blondy, Mathilde Violland, Daniel Pouliquen, Marc Grégoire, Nicolas Boisgerault, Philippe Erbs, Jean-François Fonteneau

**Affiliations:** 1 CRCINA, INSERM, Université d’Angers, Université de Nantes, 44007 Nantes, France; 2 Labex IGO, Immunology Graft Oncology, 44007 Nantes, France; 3 Transgene, 67400 Illkirch, France

**Keywords:** oncolytic immunotherapy, oncolytic virus, vaccinia virus, pleural mesothelioma, thymidine kinase, ribonucleotide reductase

## Abstract

Malignant pleural mesothelioma (MPM) is a cancer of the pleura that lacks efficient treatment. Oncolytic immunotherapy using oncolytic vaccinia virus (VV) may represent an alternative therapeutic approach for the treatment of this malignancy. Here, we studied the oncolytic activity of VV thymidine kinase (*TK*)*-*ribonucleotide reductase (*RR*)*-/*green fluorescent protein (*GFP*) against MPM. This virus is a VV from the Copenhagen strain that is deleted of two genes encoding the TK (*J2R*) and the RR (*I4L*) and that express the GFP. First, we show *in vitro* that VV*TK-RR-/GFP* efficiently infects and kills the twenty-two human MPM cell lines used in this study. We also show that the virus replicates in all eight tested MPM cell lines, however, with approximately a 10-fold difference in the amplification level from one cell line to another. Then, we studied the therapeutic efficiency of VV*TK-RR-/GFP* in non-obese diabetic (NOD) severe combined immunodeficient (SCID) mice that bear peritoneal human MPM tumors. One intraperitoneal infection of VV*TK*-*RR-/GFP* reduces the tumor burden and significantly increases mice survival compared to untreated animals. Thus, VV*TK-RR-* may be a promising oncolytic virus (OV) for the oncolytic immunotherapy of MPM.

## Graphical Abstract

**Figure:**
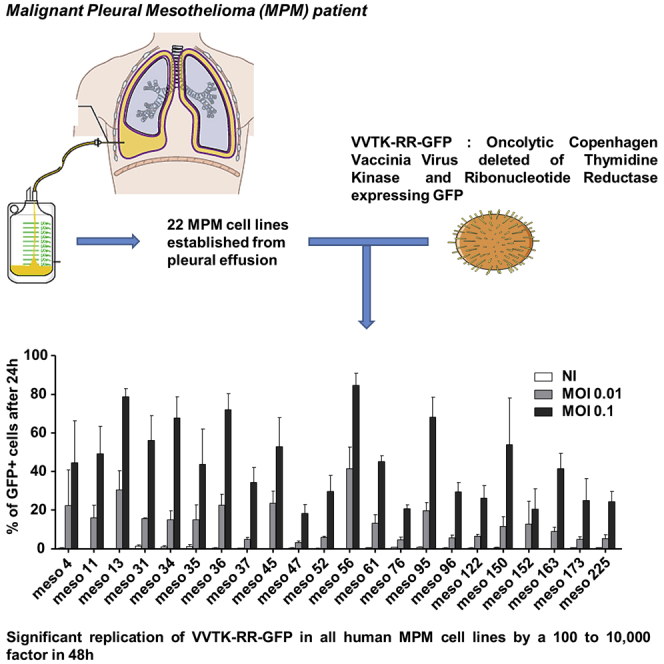

## Teaser

In this study, we show that VV*TK-RR-/GFP*, a Copenhagen vaccinia virus deleted of two virulence factors, replicates in and kills efficiently 22 human malignant pleural mesothelioma (MPM) cell lines. In mice bearing a human MPM xenograft, the VV*TK-RR-/GFP* increases its survival, highlighting the therapeutic potential of this virus against MPM.

## Introduction

Malignant pleural mesothelioma (MPM) is an aggressive tumor of the pleura, usually associated with chronic asbestos exposure, mainly during occupational activities. Incidence is increasing and is expected to peak around the year 2020 in the western world and to continue to rise in developing countries.^1^ Clinical treatments for MPM, including chemotherapy, radiotherapy, and surgery, are of limited efficacy. There is an urgent need of new therapeutic approaches.

Oncolytic virotherapy is a therapeutic strategy that is developing rapidly and recently meets its first success with approval of talimogene laherparepvec (T-vec) for treatment of metastatic melanoma.^2^ It consists in using oncolytic viruses (OVs) that exclusively or preferentially replicate in tumor cells, inducing their immunogenic cell death. Several oncolytic vaccinia viruses (VVs) are now evaluated in clinical trials for the treatment of different types of cancer.^3^ For mesothelioma, different oncolytic VVs with inactivation of the thymidine kinase (TK) gene have been studied.^4^, ^5^, ^6^, ^7^, ^8^, ^9^ These viruses exert oncolytic activities due to the high expression of *TK* in proliferating tumor cells that allows viral replication. TG6002 is a VV from the Copenhagen strain that is deleted of the *TK* and the ribonucleotide reductase (*RR*) genes and expresses the suicide gene *FCU1*.^10^ As TK, RR is a key enzyme in the supply chain of deoxyribonucleoside triphosphates (dNTPs) for DNA replication and is often overexpressed in cancer.^11^ Thus, TG6002 replicates preferentially in tumor cells due to their high TK and RR activities. This OV, in combination with 5-fluorocytosine, is entering in phase I/II clinical trials for the treatment of recurrent glioblastoma (ClinicalTrials.gov: NCT03294486) and gastrointestinal cancer (ClinicalTrials.gov: NCT03724071 and NCT04194034).

In this work, we studied a variant of TG6002 that does not contain the *FCU1* gene but instead, the gene encoding green fluorescent protein (GFP): VV*TK-RR-/GFP*, allowing a monitoring of the viral replication. We measured *in vitro* the oncolytic activity of this OV against a large panel of 22 human MPM tumor cell lines. We show that they are all lysed by VV*TK-RR-/GFP*, despite differences in the level of viral replication between tumor cell lines. We also show that VV*TK-RR-/GFP* is able to reduce *in vivo* the tumor burden and to increase significantly the survival of nonobese diabetic (NOD) severe combined immunodeficiency (SCID) mice bearing peritoneal human MPM tumors. Overall, our work shows that TG6002 may be an interesting OV for the treatment of MPM.

## Results

### MPM Cell Lines Are Highly Sensitive to the Oncolytic Activity of VV*TK-RR-/GFP In Vitro*

**Figure 1:**
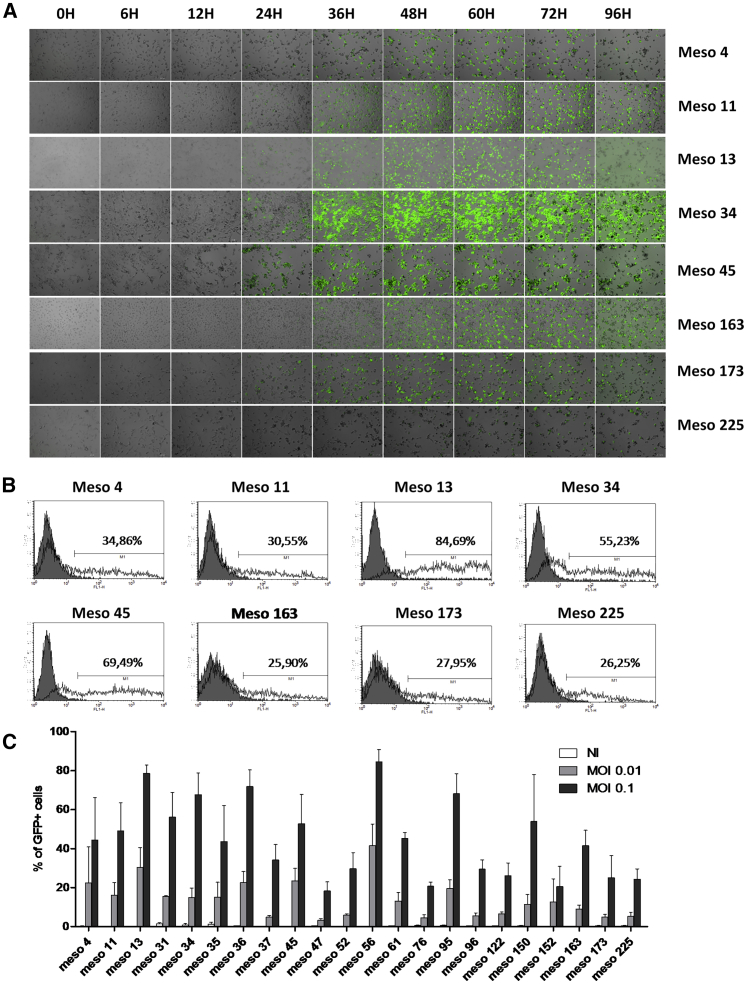
Human MPM Cell Lines Are Sensitive to VV*TK-RR-/GFP* Infection (A) Meso4, -11, -13, -34, -45, -163, -173, and -225 human MPM cell lines were cultured with VV*TK-RR-/GFP* at an MOI of 0.1 during 96 h. Pictures were taken at 0 h, 6 h, 12 h, 24 h, 36 h, 48 h, 60 h, 72 h, and 96 h. (B) Meso4, -11, -13, -34, -45, -163, -173, and -225 human MPM cell lines were cultured alone or with VV*TK-RR-/GFP* at an MOI of 0.01 or 0.1 during 24 h. GFP fluorescence was analyzed by flow cytometry. (C) 22 human MPM cell lines were cultured with VV*TK-RR-/GFP* at an MOI of 0.01 and 0.1 during 24 h. GFP fluorescence was analyzed by flow cytometry. The histogram represents mean ± SEM of three independent experiments.

First, we assessed *in vitro* the oncolytic activity of the VV*TK-RR-/GFP* against 22 human MPM cell lines established from patient pleural effusion. The *GFP* gene is under the control of a promoter active during the early and late phase of the viral replication and allowed to follow the infection during 4 days by fluorescent video microscopy (Figure 1A; Videos S1, S2, S3, S4, S5, S6, S7, and S8). We also measured the fluorescence by flow cytometry, 24 h after the infection (Figures 1B and 1C).

Video S1. Infection of Meso4 MPM Cell Line by VV*TK-RR-/GFP* Meso4 Cells were infected with VV*TK-RR-/GFP* (MOI=0.1). The time-lapse video microscopy was made using a Leica DMI6000B microscope with a 10x objective. Images were acquired every 15min for 4 days.

Video S2. Infection of Meso11 MPM Cell Line by VV*TK-RR-/GFP* Meso11 Cells were infected with VV*TK-RR-/GFP* (MOI=0.1). The time-lapse video microscopy was made using a Leica DMI6000B microscope with a 10x objective. Images were acquired every 15min for 4 days.

Video S3. Infection of Meso13 MPM Cell Line by VV*TK-RR-/GFP* Meso13 Cells were infected with VV*TK-RR-/GFP* (MOI=0.1). The time-lapse video microscopy was made using a Leica DMI6000B microscope with a 10x objective. Images were acquired every 15min for 4 days.

Video S4. Infection of Meso34 MPM Cell Line by VV*TK-RR-/GFP* Meso34 Cells were infected with VV*TK-RR-/GFP* (MOI=0.1). The time-lapse video microscopy was made using a Leica DMI6000B microscope with a 10x objective. Images were acquired every 15min for 4 days.

Video S5. Infection of Meso45 MPM Cell Line by VV*TK-RR-/GFP* Meso45 Cells were infected with VV*TK-RR-/GFP* (MOI=0.1). The time-lapse video microscopy was made using a Leica DMI6000B microscope with a 10x objective. Images were acquired every 15min for 4 days.

Video S6. Infection of Meso163 MPM Cell Line by VV*TK-RR-/GFP* Meso163 Cells were infected with VV*TK-RR-/GFP* (MOI=0.1). The time-lapse video microscopy was made using a Leica DMI6000B microscope with a 10x objective. Images were acquired every 15min for 4 days.

Video S7. Infection of Meso173 MPM Cell Line by VV*TK-RR-/GFP* Meso173 Cells were infected with VV*TK-RR-/GFP* (MOI=0.1). The time-lapse video microscopy was made using a Leica DMI6000B microscope with a 10x objective. Images were acquired every 15min for 4 days.

Video S8. Infection of Meso225 MPM Cell Line by VV*TK-RR-/GFP* Meso225 Cells were infected with VV*TK-RR-/GFP* (MOI=0.1). The time-lapse video microscopy was made using a Leica DMI6000B microscope with a 10x objective. Images were acquired every 15min for 4 days.

We found that all MPM cell lines were sensitive to the infection by VV*TK-RR-/GFP*. The first wave of green fluorescent tumor cells usually appears between 12 h and 15 h after the start of the infection. After 24 h, the infection spreads to other cells with the apparition of a second wave of green fluorescent tumor cells. Between 24 h and 48 h after the infection, the mobility of tumor cells decreases. This slowing down is quite impressive with Meso11, Meso13, and Meso163 MPM cell lines (Videos S2, S3, and S6). After 48 h, tumor cells detached from the plastic support and get a round shape, probably due to apoptosis induction. Tumor cells then start to burst. Ruptures of the cell membrane provoke rapid loss of the green fluorescence. This secondary necrosis is more or less rapid with cell lines, like Meso13, that have lost all of the fluorescence by 96 h, and cell lines that take more time to burst, such as Meso34 (Videos S3 and S4). After 4 days, all of the 22 MPM cell lines infected by VV*TK-RR-/GFP* exhibit morbidity that leads to cell death in a few more days.

During the infection, the level of maximum fluorescence varied from one cell line to another. For instance, Meso225 and Meso173 failed to reach a high level of fluorescence, whereas Meso13 and Meso34 reached a high level of fluorescence (Figure 1B; Videos S3, S4, S7, and S8). Altogether, these results show that human MPM is highly sensitive to VV*TK-RR-/GFP* infection.

**Figure 2:**
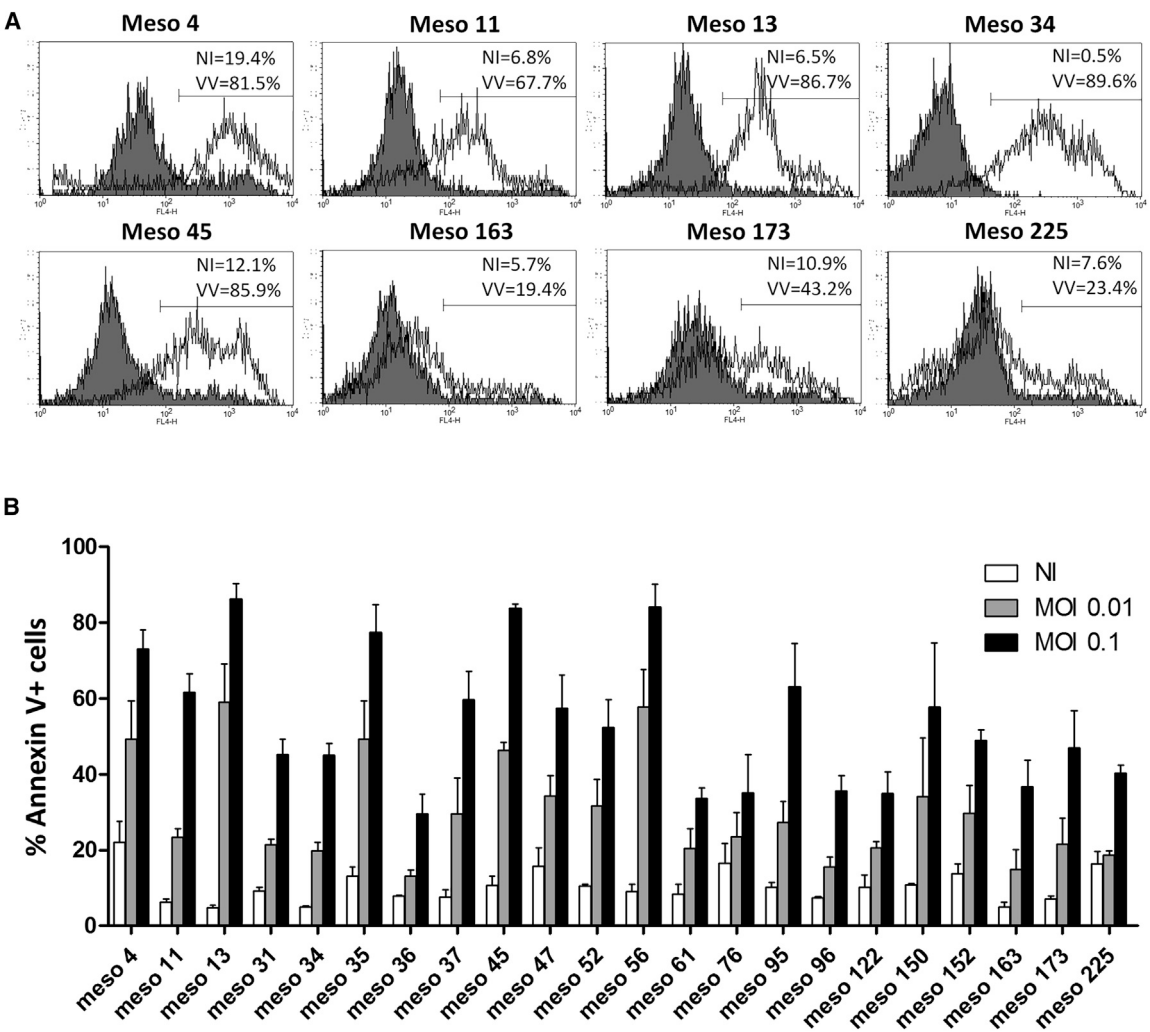
Human MPM Cell Lines Are Sensitive to VV*TK-RR-/GFP* Oncolytic Activity (A) Meso4, -11, -13, -34, -45, -163, -173, and -225 human MPM cell lines were cultured alone or with VV*TK-RR-/GFP* at an MOI of 0.1 during 48 h. Cells were labeled with Annexin V-APC and propidium iodide. Annexin V-APC fluorescence was analyzed by flow cytometry. Gray histograms represent cells cultured alone and white histograms, cells cultured with VV*TK-RR-/GFP*. (B) 22 human MPM cell lines were cultured with VV*TK-RR-/GFP* at an MOI of 0.01 and 0.1 during 48 h. Cells were labeled with Annexin V-APC and propidium iodide. Annexin V-APC fluorescence was analyzed by flow cytometry. The Histogram represents mean ± SEM of three independent experiments.

Then, we studied the induction of cell death by VV*TK-RR-/GFP* on the 22 MPM cell lines by Annexin V-allophycocyanin (APC)/propidium iodide (PI) staining, 48 h after infection. The GFP fluorescence from the infected cells was so strong that it interferes with the PI staining and could not be compensated. However, we observed a significant Annexin V staining on the 22 MPM cell lines in the presence of VV*TK-RR-/GFP* (Figure 2). It confirms the observations made by video microscopy (Videos S1, S2, S3, S4, S5, S6, S7, and S8) that all tested human MPM cell lines are sensitive to the oncolytic activity of VV*TK-RR-/GFP*.

**Figure 3:**
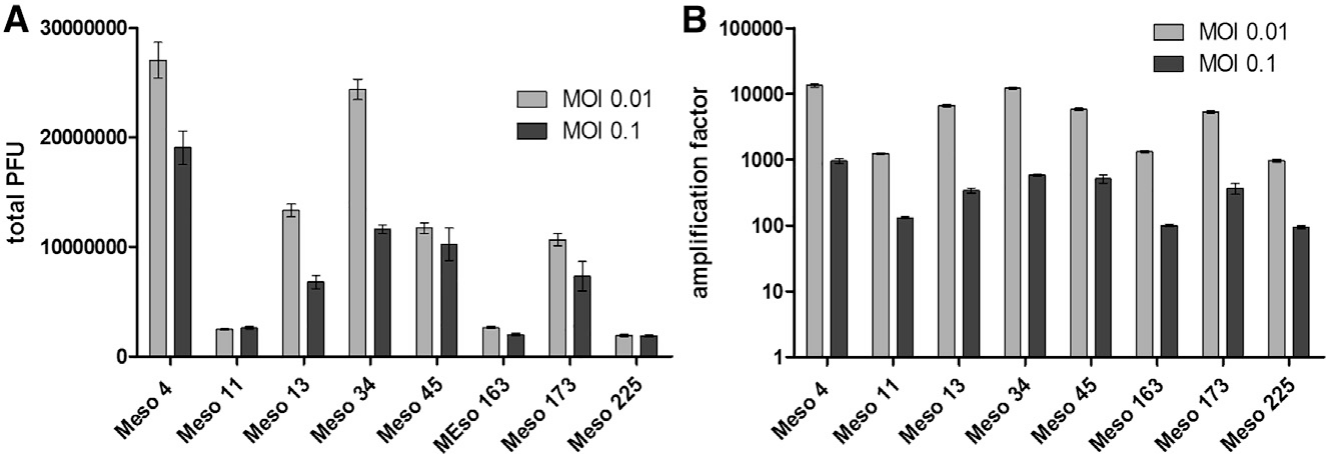
Human MPM Cell Lines Are Permissive to VV*TK-RR-/GFP* Replication Meso4, -11, -13, -34, -45, -163, -173, and -225 human MPM cell lines were cultured with VV*TK-RR-/GFP* at an MOI of 0.01 and 0.1 during 48 h. Culture supernatants and cells were then collected, and the presence of the VV*TK-RR-/GFP* infectious particle was measured by PFU assay. (A) The histogram represents the total PFUs found in cells and supernatants. (B) The histogram represents the amplification factor determined by dividing the total produced PFUs by the PFUs used at the start of the experiments. The histograms represent mean ± SEM of three independent experiments.

Then, we measured the production of VV*TK-RR-/GFP* in 8 MPM cell lines, 48 h postinfection. We were able to detect replication of the virus in all MPM cell lines tested (Figure 3A), especially at the lowest MOI (0.01). Replication was low in three MPM cell lines (Meso11, -163, and -225), medium in three others (Meso13, -45, and -173), and high in the last two (Meso4 and -34). Replication was higher at the lowest MOI (0.01), since it allows more wave of infections than the highest MOI (0.1) that exerts cytopathic effects (Figure 3B). Thus, we found production of infectious viral particles in the 8 tested MPM cell lines, highlighting the capacity of this virus to replicate in MPM cells.

### *In Vivo* Oncolytic Activity of VV*TK-RR-/GFP*

**Figure 4:**
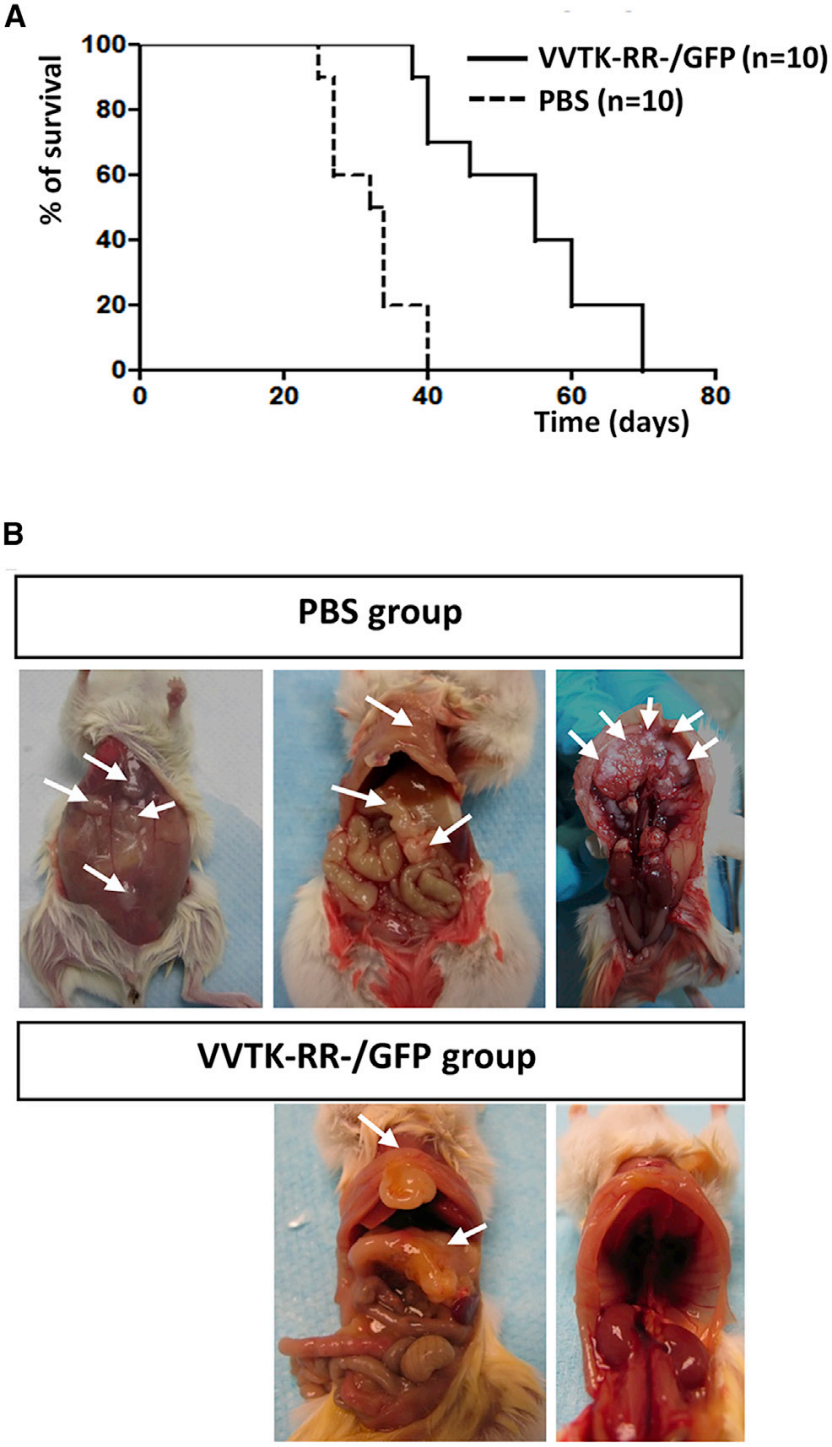
One Intraperitoneal Injection of VV*TK-RR-/GFP* Increases Survival of NOD SCID Mice Engrafted with the Human MPM Cell Line Meso163 and Decreases Tumor Formations NOD SCID mice were challenged intraperitoneally with 5 × 10^6^ Meso163 human MPM cells. After 3 weeks, at day 0, PBS or VV*TK-RR-/GFP* (1 × 10^7^ PFU) was injected intraperitoneally. (A) The graph represents survival of NOD SCID mice. (B) Autopsies of representative NOD SCID mice engrafted with the human MPM cell line Meso163 and treated with PBS or VV*TK-RR-/GFP*. Arrows indicate tumors.

To confirm our *in vitro* observations regarding oncolytic activity of VV*TK-RR-/GFP*, we evaluated the virus for the treatment of NOD SCID mice that bear an intraperitoneal human Meso163 tumor (Figure 4). In a first experiment on a small number of NOD SCID mice (n = 3), we determined that the injection of 1 × 10^7^ plaque-forming units (PFUs) was not toxic, whereas 1 × 10^8^ PFUs were lethal (data not shown). In a second experiment, we observed that the intraperitoneal injection of VV*TK-RR-/GFP* to NOD SCID mice that bear Meso163 tumor cells significantly increased the mean survival (MS) compared to mice that received phosphate-buffered saline (PBS; MS PBS = 33 days; MS VV*TK-RR-/GFP* = 55 days; p value = 0.0001 [Mantel-Cox]) (Figure 4A).

Mice were dissected to evaluate the tumor burden (Figure 4B). Mice that received PBS exhibit numerous metastasis on the abdominal muscular layer, the omentum, the mesentery, the stomach, the peritoneum, and the diaphragm. In VV*TK-RR-/GFP*-treated animals, we observed not more than three massive tumors that often develop in the omentum and/or near the sternum in all animals. These results suggest that VV*TK-RR-/GFP* succeeded to eradicate a lot of metastasis but failed to control a very small number of tumors that may be the primary tumors.

## Discussion

Altogether, these results show that all human MPM cell lines are highly sensitive to the oncolytic activity of VV*TK-RR-/GFP* from the Copenhagen strain. *In vitro*, the virus is able to replicate and induces cell death that ends up into secondary necrosis. Another study addressed the *in vitro* sensitivity of six human MPM cell lines to the GLV-1h68, an oncolytic VV from the Lister strain, that is deleted of TK.^6^ It reported that all MPM cell lines were sensitive. In a previous study, Mukherjee et al.^5^ showed that two human MPM cell lines were sensitive to the NYCBOH strain of VV deleted of TK and encoding interleukin (IL)-2. Herein, we show that 22 out 22 human MPM cell lines are sensitive. This result also suggests that the antiviral type I interferon (IFN I) response seems to fail to protect tumor cells from VV*TK-RR-/GFP* replication. Indeed, we recently showed on the same 22 human MPM cell lines that 15 exhibit defects in the IFN I response that makes them sensitive to the oncolytic-attenuated measles virus.^12^ 7 MPM cell lines were able to control measles virus replication by a functional IFN I response. Among these 7 MPM cell lines , we showed that Meso4, -45, and -173 are not able to control VV*TK-RR-/GFP* replication. Thus, either VV*TK-RR-/GFP* replication is not sensitive to the type I IFN response due to virulence factors, such as B18R, that are able to block the type I IFN response,^13^ or there is a failure to detect DNA viruses and thus, the VV*TK-RR-/GFP* by these cell lines to induce a type I IFN response.

We also show in this study that a single intraperitoneal injection of VV*TK-RR-/GFP* to immunodeficient mice engrafted with a human MPM cell line increases significantly their survival and reduces the spreading of metastasis. Therapeutic benefits of different VVs deleted of the *TK* gene have been reported in human mesothelioma xenograft mouse models,^6^^,^^8^ as well as in syngeneic mesothelioma mouse models.^14^, ^15^, ^16^ The failure of VV*TK-RR-/GFP* to totally cure the mice in our study may be due to the absence of an adaptive immune system that should enhance the oncolytic efficacy by the induction of an antitumor immune response. Indeed, it has been shown that oncolytic VVs induce an immunogenic cell death that allows antigen-presenting cells to prime naive T cells to develop an antitumor immune response.^17^^,^^18^ We also reported recently that VV*TK-RR-/GFP* can increase effector cytotoxic CD4^+^ T cell activation by increasing the human leukocyte antigen (HLA) class II tumor antigen presentation by tumor cells.^19^

Phase I clinical trials of mesothelioma treatment by oncolytic VV deleted of the *TK* gene and encoding IL-2 have demonstrated that this approach is not toxic but lacks therapeutic efficacy.^4^^,^^5^ Other modifications of the oncolytic VV may be necessary to increase its efficacy, such as the addition of the gene encoding CXCL11^14^^,^^15^ Furthermore, VVs can be armed with the suicide gene *FCU1*, encoding an enzyme that transforms locally in the tumor the prodrug 5-fluorocytosine into the toxic drug 5-fluorouracile.^10^^,^^20^ The virus VV*TK-RR-Fcu1+* (TG6002) is now evaluated in phase I/II clinical trials for the treatment of gastrointestinal cancers in combination with 5-fluorocytosine (ClinicalTrials.gov: NCT03724071 and NCT04194034) and of recurrent glioblastoma (ClinicalTrials.gov: NCT03294486). Given that MPM is resistant to all conventional treatments, the oncolytic VV may represent a promising therapeutic approach.

## Materials and Methods

### Cell Culture

The 22 human MPM cell lines (from Meso4 to Meso225) were established in our laboratory from pleural effusions collected by thoracocentesis and genetically characterized.^21^ All patients gave their informed consent. All cell lines were maintained in RPMI-1640 medium, supplemented with 10% heat-inactivated fetal calf serum, 100 U/mL penicillin, 100 μg/mL streptomycin, and 2 mM L-glutamine (all reagents from Gibco-Invitrogen), and cultured at 37°C in a 5% CO_2_ atmosphere. Cells were routinely checked for Mycoplasma contamination using the PlasmoTest from InvivoGen.

### Vaccinia virus VVT*K-RR-/GFP*

Attenuated recombinant vaccinia virus VV*TK-RR-/GFP* was derived from the Copenhagen strain and was deleted in the TK and RR genes and expressed the GFP. GFP is under the control of the p11k7.5 early-late promoter.^20^ VV*TK-RR-/GFP* was propagated and titrated in chicken embryo fibroblasts, as previously described.^22^

### Video Microscopy

1 day before infection, MPM cells were seeded in 24-well plates at a density of 10^5^ cells/well. Cells were then infected with VV*TK-RR-/GFP* (MOI = 0.1). The time-lapse video microscopy was made using a Leica DMI6000B microscope with a 10× objective. Images were acquired every 15 min for 4 days. We used MetaMorph Microscopy Automation and Image Analysis Software (version 7.8) and Fiji software for acquisition and analysis.^23^

### Measurement of MPM Cell Line Infection by VV*TK-RR-/GFP*

MPM cell lines were seeded in 12-well plates at a density of 0.2 × 10^6^ cells/well in 2 mL of culture medium containing VV*TK-RR-/GFP* at an MOI of 0.01 or 0.1. After 24 h of culture, cells were harvested for GFP fluorescence measurement. Cells were fixed with PBS containing 1% paraformaldehyde during 10 min at room temperature and washed with PBS. GFP fluorescence was measured by flow cytometry on a FACSCalibur (BD Biosciences) and analyzed using the BD CellQuest Pro software.

### Measurement of MPM Cell Death

To measure MPM cell death induced by VV*TK-RR-/GFP*, MPM cell lines were cultured and exposed to VV*TK-RR-/GFP* as for the measurement of infection above. After 48 h, some supernatants were collected for measurement of VV*TK-RR-/GFP* replication below, and cells were harvested and labeled with an Annexin V-APC/PI labeling kit (BD Pharmingen), according to the manufacturer’s instructions. Cells were then fixed with PBS 1% paraformaldehyde. Annexin V-APC fluorescence was analyzed by flow cytometry as above.

### Measurement of VV*TK-RR-/GFP* Replication in MPM Cell Lines

A PFU assay was performed on supernatants and cells collected from the 48-h cultures of MPM cell lines with VV*TK-RR-/GFP*, as described.^22^ The VV*TK-RR-/GFP* amplification factor was determined by dividing the PFU found in the supernatants by the PFUs that were added at the start of the culture.

### VV*TK-RR-/GFP* Treatment of NOD SCID Mice Bearing Human MPM Meso163 Cell Line Xenograft

All *in vivo* experiments complied with European Union regulations on the welfare and use of animals in cancer research. 6-week-old female NOD SCID mice were purchased from Centre d’Elevage Janvier (Le Genest-Saint-Isle, France). Twenty mice were challenged intraperitoneally with 5 × 10^6^ tumor cells. After 3 weeks, 100 μL of PBS was injected in the intraperitoneal cavity of one-half of the mice. The others received 100 μL of PBS containing 1 × 10^7^ PFUs of VV*TK-RR-/GFP* intraperitoneally. Mice were followed at least every 2 days and sacrificed when they became moribund (weight loss, decreased mobility). Mice were then dissected and pictured.

## Author Contributions

Experiment Conception, J.-F.F. and P.E.; Experiments, T.D., J.N., M.G., I.F., V.H., J.F., T.B., and M.V.; Data Analysis and Manuscript Writing, M.G., N.B., P.E., and J.-F.F.

## Conflicts of Interest

The authors declare no competing interests.
